# Supplementary material for: Structural basis for immune cell binding of Fusobacterium nucleatum via the trimeric autotransporter adhesin CbpF
Source: Proc Natl Acad Sci U S A. 2025 Apr 8;122(15):e2418155122. doi: 10.1073/pnas.2418155122 (PMC12012533; doi:10.1073/pnas.2418155122)
Supplement: Supplementary file 1 — Appendix 01 (PDF) [file pnas.2418155122.sapp.pdf]

Supporting Information for

Structural basis for immune cell binding of *Fusobacterium nucleatum* via the trimeric autotransporter adhesin CbpF

Gian Luca Marongiu[1,3], Uwe Fink[1,3], Felix Schöpf[1], Andreas Oder[2], Jens Peter von Kries[2], Daniel Roderer[1]*

1: Leibniz-Forschungsinstitut fur Molekulare Pharmakologie, Robert-Roessle-Str. 10, 13125 Berlin, Germany.

2: Leibniz-Forschungsinstitut fur Molekulare Pharmakologie, Screening Unit, Robert-Roessle-Str. 10, 13125 Berlin, Germany.

3: These authors contributed equally.

*Corresponding author:

Daniel Roderer

email: roderer@fmp-berlin.de

This PDF file includes:

Figures S1 to S10

Tables S1 to S2

Movie S1 legend

Supplementary Figures

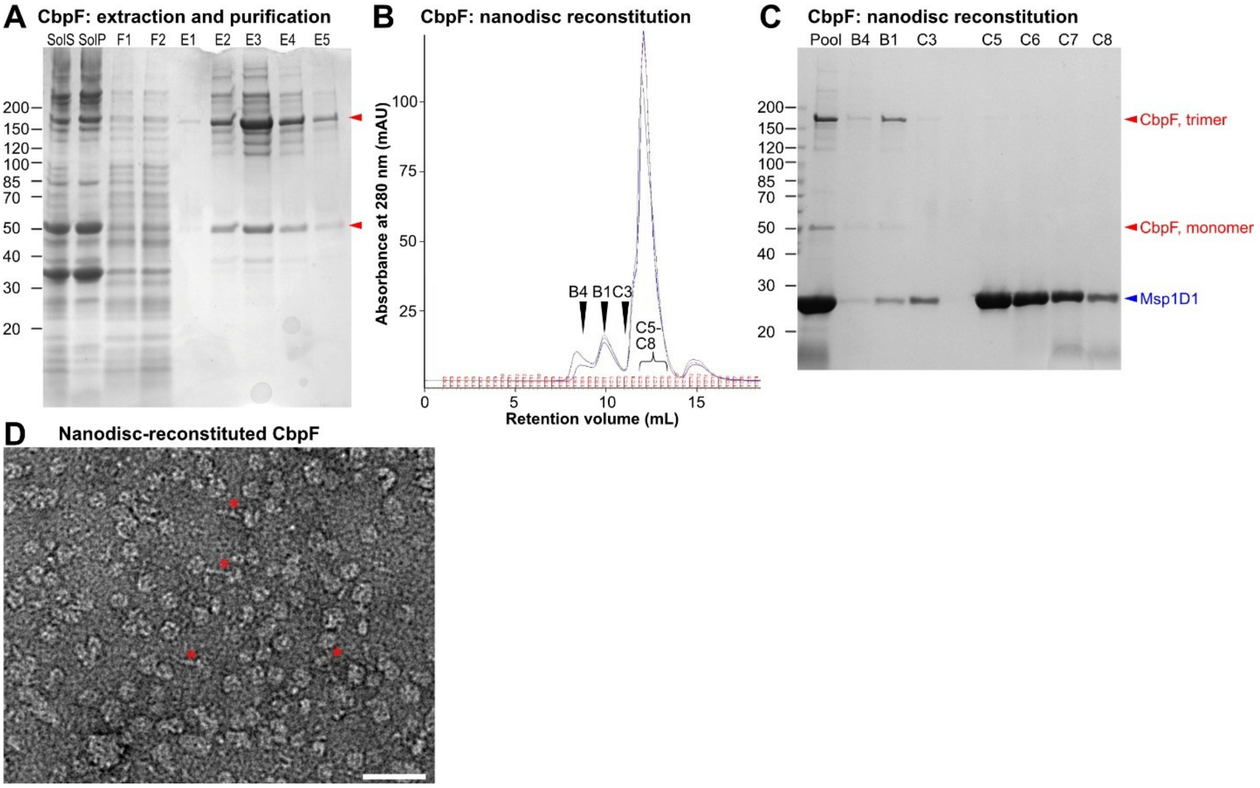

Figure S1: Purification and nanodisc reconstitution of CbpF from *F. nucleatum* ATCC25586. **A:** SDS-PAGE showing the purification of membrane-extracted CbpF using StrepTactin resin. SolS/SolP: supernatant and pellet after solubilization, F1, F2: flowthrough fractions 1 and 2, E1-E5: elution fractions. Red arrowheads indicate CbpF monomer at 50 kDa and trimer at 150 kDa, respectively. Note the formation of an SDS-resistant and heat-resistant trimer, which is noncovalently formed due to the absence of cysteines in CbpF. **B:** Chromatogram of CbpF after nanodisc reconstitution (CbpF-to-MSP1D1-to-lipid ratio of 1:5:80) on a Superdex 200 increase 10/300 column. The three chromatograms show three consecutive runs of a reconstituted sample, where 500 µL were loaded each. The fractions shown in C are indicated. **C:** SDS-PAGE showing nanodisc reconstitution of CbpF and purification via size exclusion chromatography. The fractions on the gel are indicated in B. **D:** Section of a negative stain EM micrograph of nanodisc-reconstituted CbpF (fraction B1 of B,C), recorded at 120 kV. The red asterisks indicate rod-shaped CbpF that protrudes from nanodiscs. Scale bar: 50 nm.

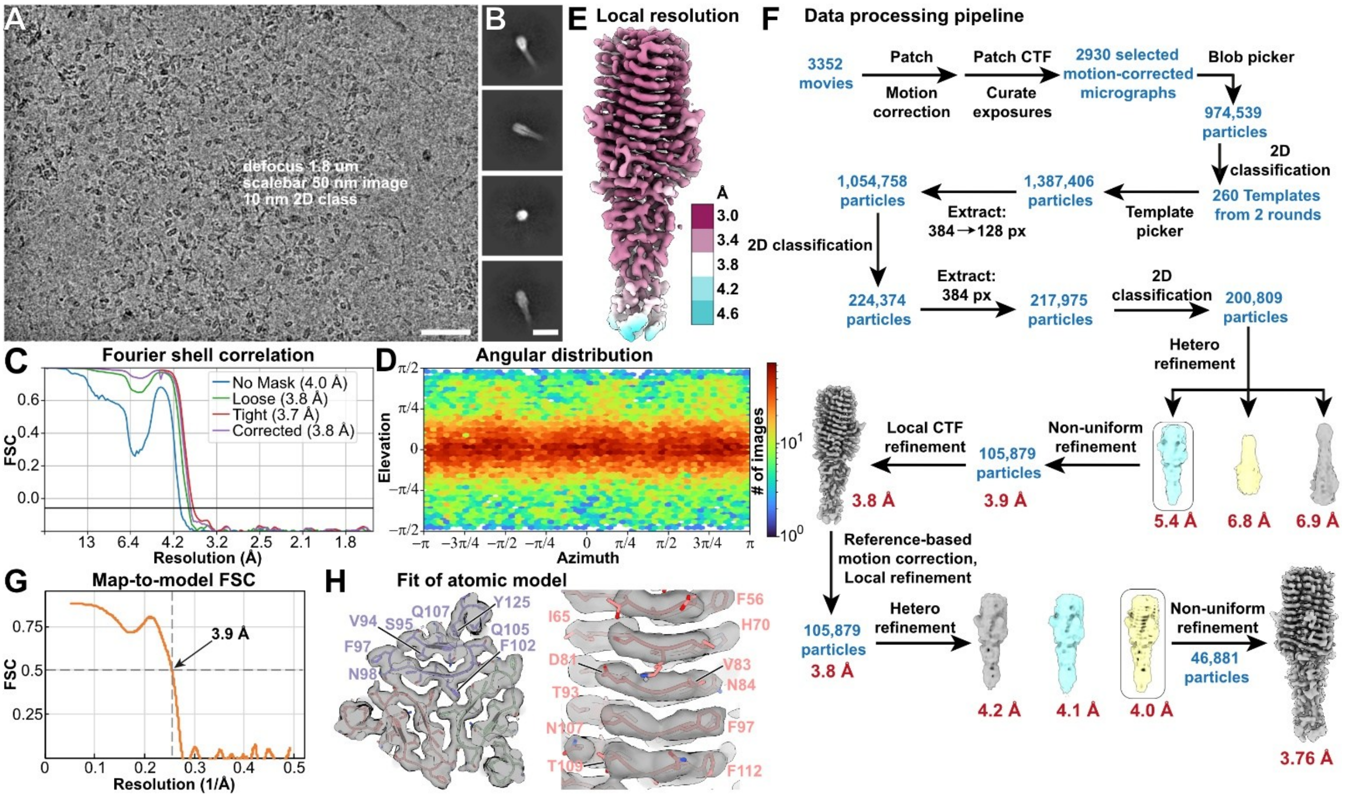

Figure S2: Cryo-EM and SPA of CbpF. A: Representative cryo-EM micrograph recorded at 300 kV and -1.8 µm defocus. Scale bar: 50 nm. **B:** Representative 2D class averages, showing the membrane-distal domain of CbpF in side views and top view. Scale bar: 10 nm. **C,D,E:** Fourier shell correlation (C), angular distribution (D), and density map colored by local resolution (E) of the final cryo-EM density map of CbpF from 46,881 particles with C3 symmetry applied. **F:** SPA data processing scheme applied for CbpF. All steps were carried out in cryoSPARC, and all refinements after the first Hetero refinement were carried out with C3 symmetry. **G:** Map-to-model correlation of the CbpF model (residues 3-252) against the final non-sharpened density map. **H:** Fit of atomic model in selected parts of the density map. Residues for one of the three protomers are indicated. The density maps in E, H, and those in F derived from non-uniform refinements have been sharpened with DeepEMhancer.

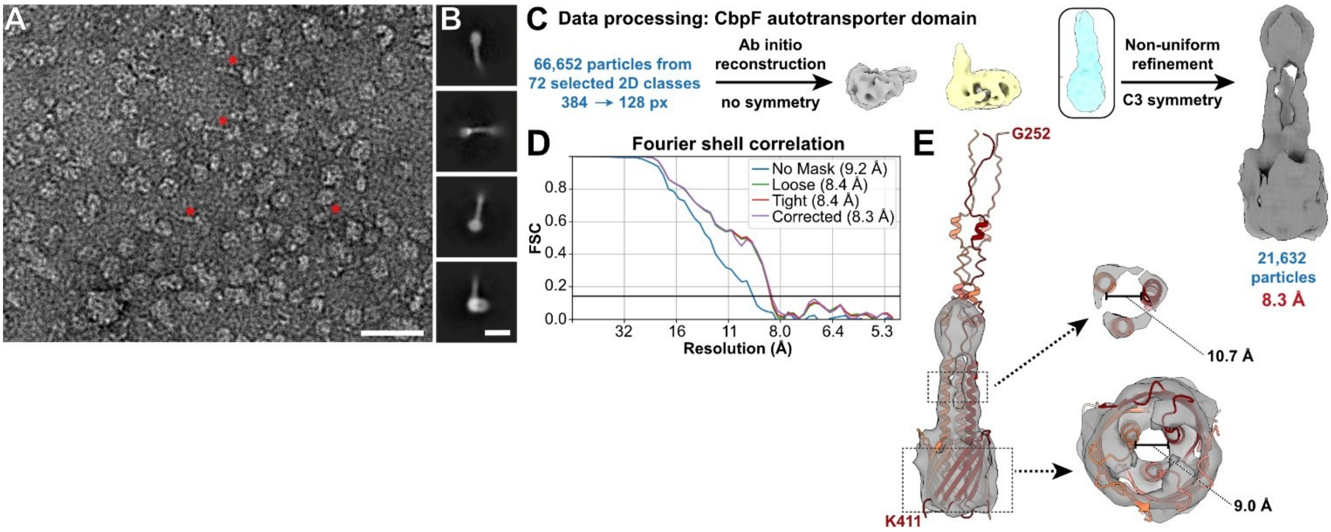

Figure S3: The autotransporter domain of CbpF. A: Representative 2D class averages that show nanodisc density with protruding CbpF. The class averages originate from the cryo-EM data as in Fig. S2. Scale bar: 10 nm. **B:** Data processing of particles underlying the 2D classes that show nanodisc-embedded CbpF autotransporter domains. **C:** Fourier shell correlation of the density map of the CbpF autotransporter domain, originating from 21,632 particles and C3 symmetry. **D:** Fit of the AlphaFold2 prediction of CbpF (residues 274 - 433) to the autotransporter density map. The three protomers were fitted independently. Inner diameters within the autotransporter domain and between the three protruding helices are shown.

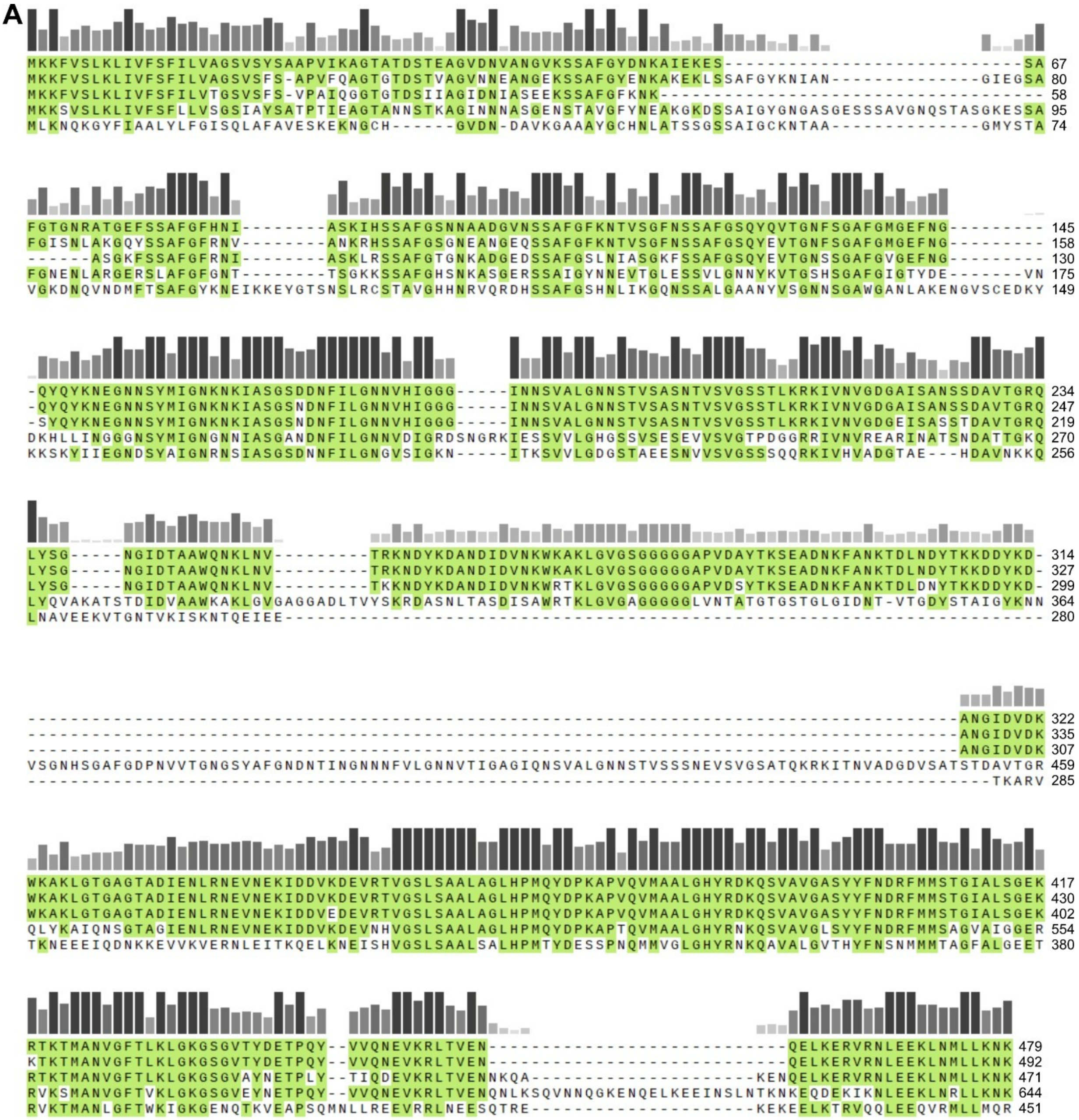

B

Percent identity matrix

Fn 25586	100.00%	93.93%	88.58%	60.67%	42.08%
Fn 23726	93.93%	100.00%	89.01%	60.49%	41.49%
F. v.	88.58%	89.01%	100.00%	59.15%	41.16%
F. p.	60.67%	60.49%	59.15%	100.00%	38.25%
F. nec.	42.08%	41.49%	41.16%	38.25%	100.00%

Figure S4: Comparison of CbpF in *Fusobacterium nucleatum* strains ATCC25586, ATCC23726, *F. vincentii*, *F. polymorphum*, and *F. necrophorum*. A: Full sequence alignment from which the excerpt in Fig. 2A is derived. The order of sequences from top to bottom is as indicated above. **B:** Identity matrix of the five Fn sequences.

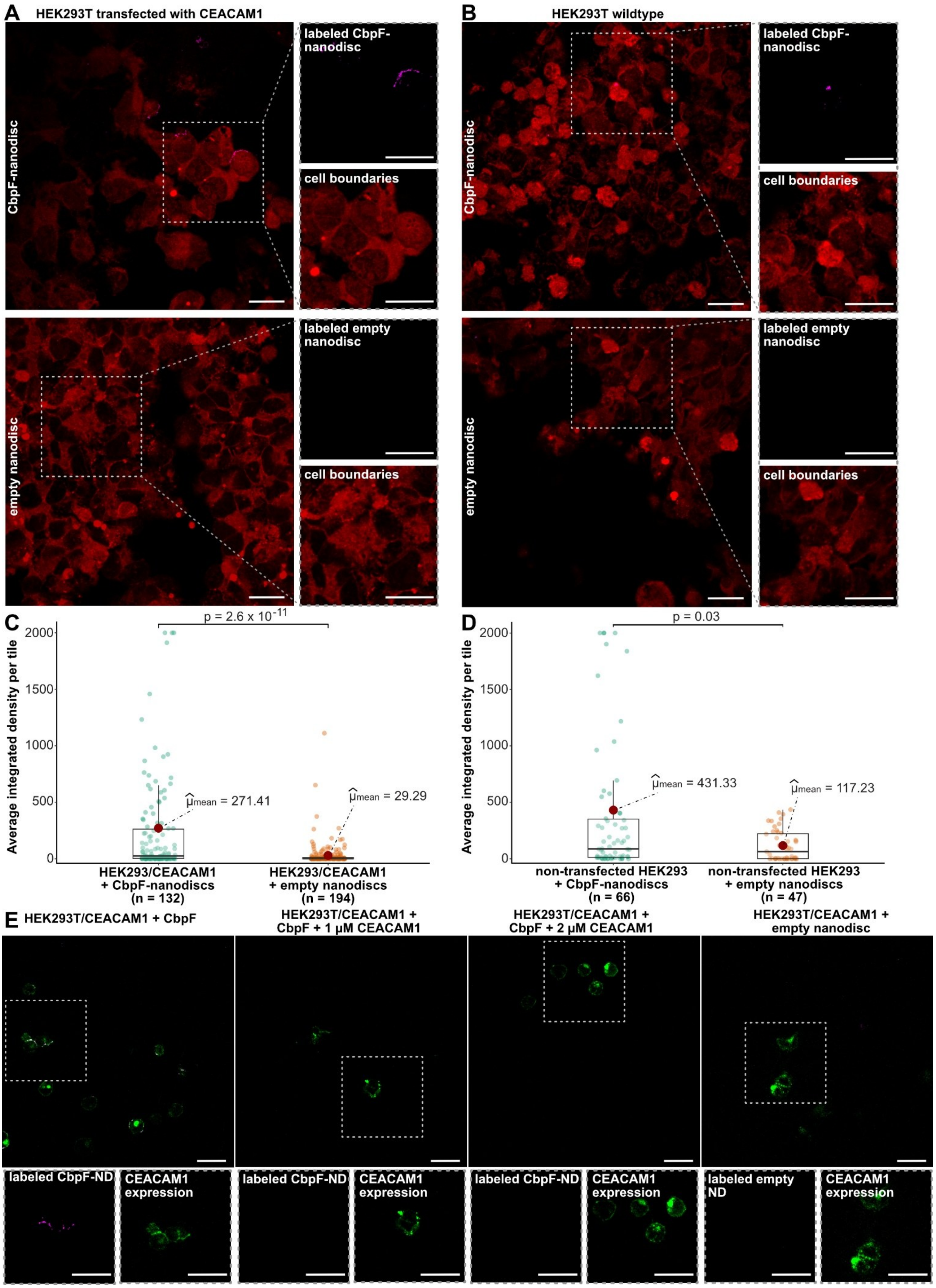
A HEK293T transfected with CEACAM1
CbpF-nanodisc
labeled CbpF-nanodisc
cell boundaries
empty nanodisc
labeled empty nanodisc
cell boundaries
B HEK293T wildtype
CbpF-nanodisc
labeled CbpF-nanodisc
cell boundaries
empty nanodisc
labeled empty nanodisc
cell boundaries
C
p = 2.6 x 10^-11
Average integrated density per tile
2000
1500
1000
500
0
μ̂mean = 271.41
μ̂mean = 29.29
HEK293/CEACAM1 + CbpF-nanodiscs (n = 132)
HEK293/CEACAM1 + empty nanodiscs (n = 194)
D
p = 0.03
Average integrated density per tile
μ̂mean = 431.33
μ̂mean = 117.23
non-transfected HEK293 + CbpF-nanodiscs (n = 66)
non-transfected HEK293 + empty nanodiscs (n = 47)
E HEK293T/CEACAM1 + CbpF
HEK293T/CEACAM1 + CbpF + 1 μM CEACAM1
HEK293T/CEACAM1 + CbpF + 2 μM CEACAM1
HEK293T/CEACAM1 + empty nanodisc
labeled CbpF-ND
CEACAM1 expression
labeled CbpF-ND
CEACAM1 expression
labeled CbpF-ND
CEACAM1 expression
labeled empty ND
CEACAM1 expression

Figure S5: Binding of CbpF to CEACAM1-expressing cells. A,B: Representative micrographs that illustrate binding of nanodisc-embedded CbpF (upper panels) in comparison to empty nanodiscs (lower panels) to human embryonic kidney (HEK293T) cells that have been transfected with a CEACAM1 expression plasmid (**A**) or not (wildtype, **B**). Magenta channel: protein labeled with AF647 red channel: cell boundaries labeled with CellBrite Fix 555 Membrane Stain. Scale bar: 25 µm. **C,D:** Quantification of experiments as in A,B, with average fluorescence signal of protein bound to cells determined from the indicated number of micrographs each. The mean is shown as red dot. The boxes represent the interquartile range, with the median shown as horizontal line within the box. Data was visualized with the ggstatsplot package (Patil, 2021) and statistical analysis was performed using the ggsignif package in R (Ahlmann-Eltze and Patil, 2021). **E**: Representative micrographs that illustrate binding of nanodisc-embedded CbpF with or without the presence of the indicated concentrations of purified CEACAM1-ECD to HEK293T cells that have been transfected with a CEACAM1 expression plasmid. Magenta channel: protein labeled with AF 647, green channel: fluorescence of CEACAM1-eGFP fusion protein. Scale bar: 25 µm. Quantification of data in Fig. 3C.

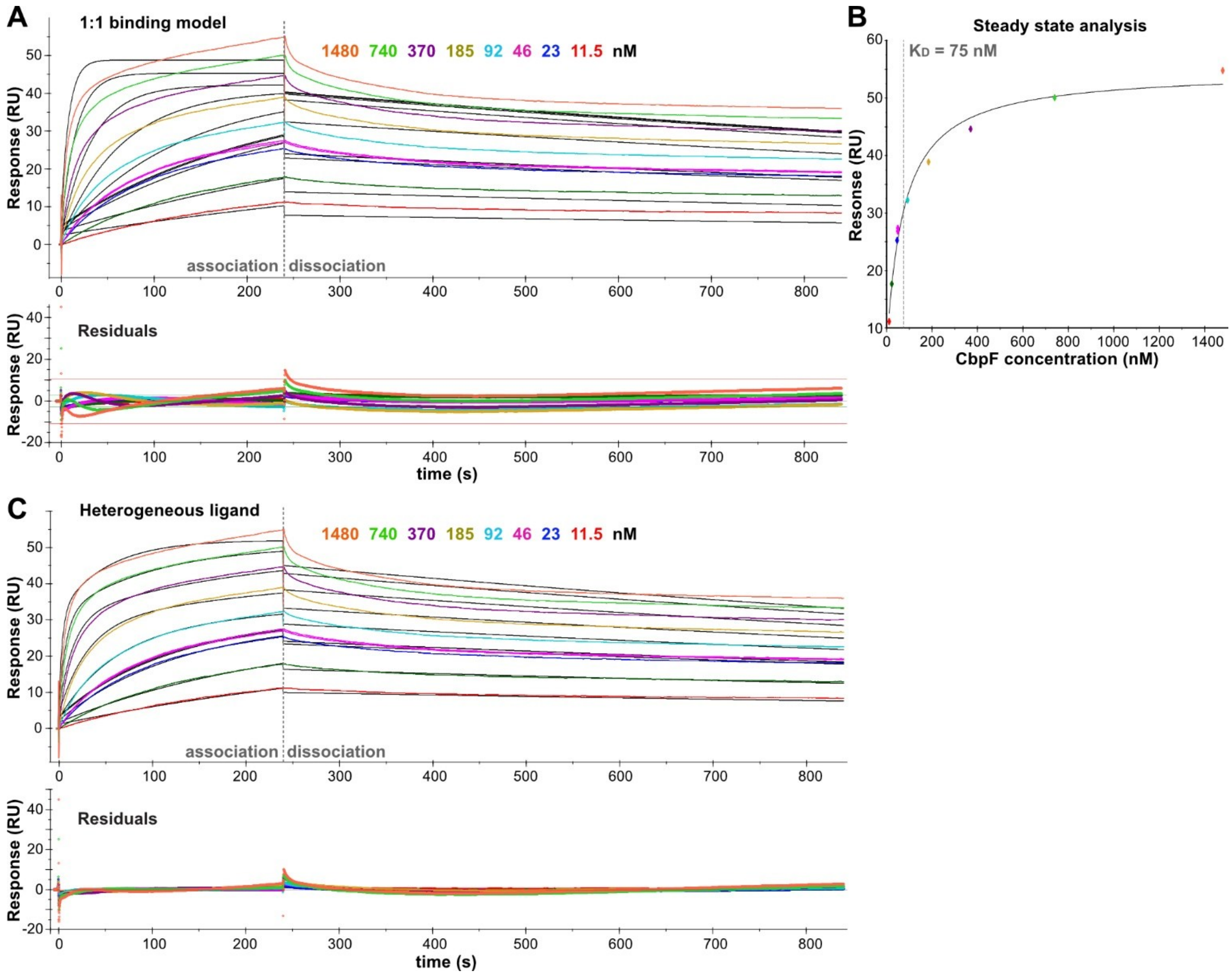

Figure S6: SPR of CbpF and CEACAM1. A: SPR sensorgram of CbpF and immobilized CEACAM1-ECD, as in Fig. 3D, with CbpF concentrations indicated. The black solid lines show a global fit of the binding curves according to a 1:1 binding model. The lower panel shows the residuals of the fit. **B:** Steady-state affinity of the SPR data as shown in Fig. 3D. The fit revealed a K_D of 75 ± 14 nM. **C:** SPR sensorgram as in C with the data fitted according to a heterogeneous ligand model. K_D values of 2.5 and 47 nM were obtained. The lower panel shows the residuals of the fit.

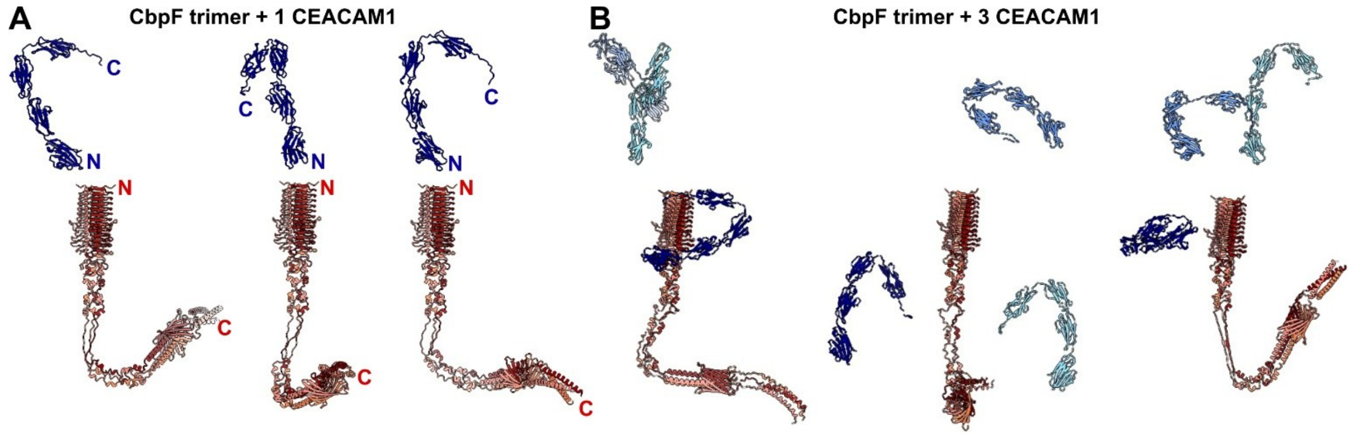

Figure S7: Representative AlphaFold2 complex predictions of CbpF (shades of red) and CEACAM1 (shades of blue). A: Three highest-rated predictions of 3:1 complex. N- and C-termini of CEACAM1 and one CbpF within the trimer are indicated. **B:** Three highest-rated predictions of 3:3 complex. No meaningful and reproducible CbpF/CEACAM1 complexes could be predicted in both cases.

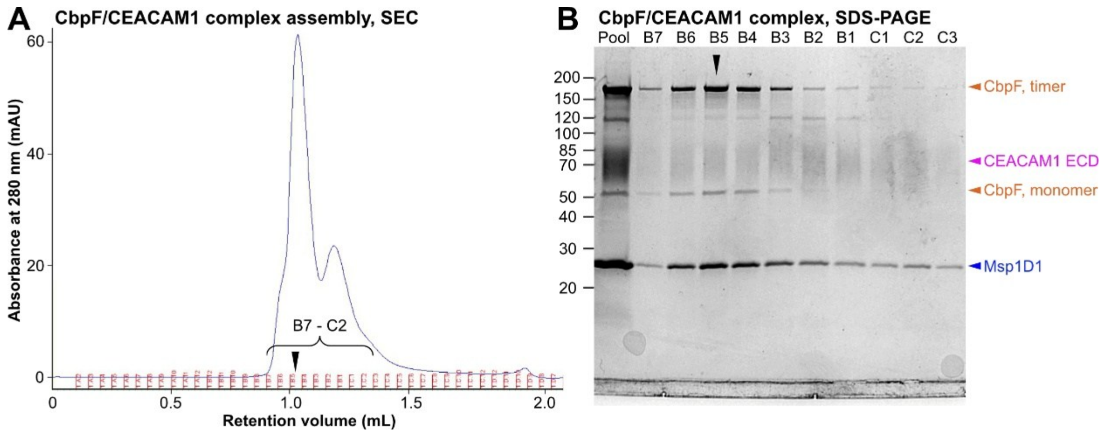

Figure S8: Assembly of nanodisc-reconstituted CbpF with CEACAM1-ECD. A: Chromatogram of CbpF/CEACAM1 (1:1 molar ratio) on a Superdex 200 increase 3.2/300 column. The fractions shown in B are indicated. **B:** SDS-PAGE showing fractions of SEC as in A. Bands corresponding to CbpF, CEACAM1 and the nanodisc scaffold protein MSP1D1 are indicated by orange, magenta, and blue arrowheads. The fraction B5 used for cryo-EM is indicated by a black arrowhead in A and B. Note the faint blurry bands that correspond to CEACAM1-ECD, indicative for a glycosylated protein and in agreement with the manufacturer's analysis.

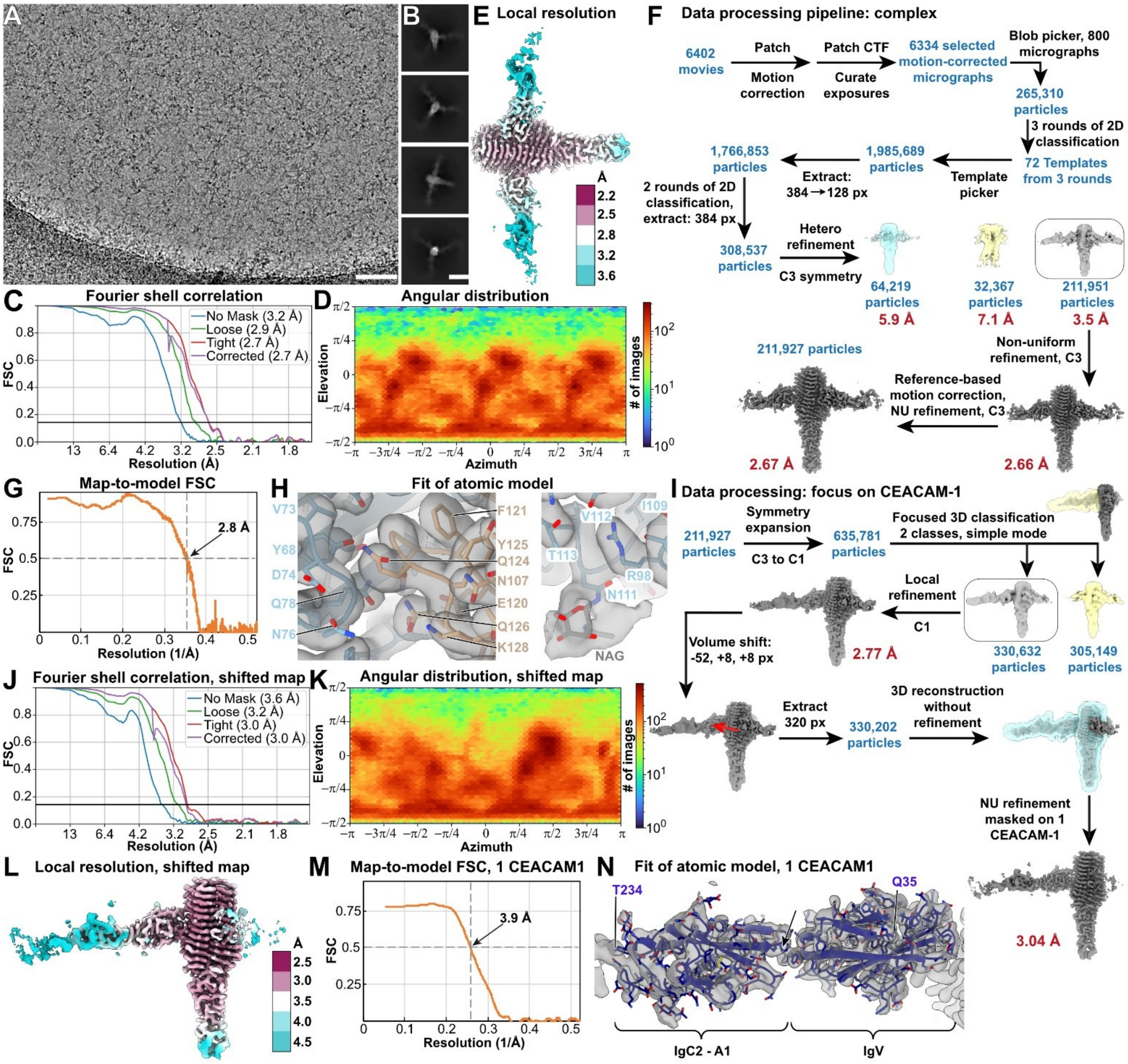

Figure S9: Cryo-EM and SPA of CbpF/CEACAM1 complex. A: Representative cryo-EM micrograph recorded at 300 kV and -1.5 µm defocus. Scale bar: 50 nm. **B:** Representative 2D class averages, showing different views of the complex. Scale bar: 10 nm. **C,D,E:** Fourier shell correlation (C), angular distribution (D), and density map colored by local resolution (E) of the final cryo-EM density map of the complex from 211,927 particles with C3 symmetry applied. **F:** SPA data processing scheme applied for the complex. All steps were carried out in cryoSPARC, and all refinements were carried out with C3 symmetry. **G:** Map-to-model correlation of the CbpF/CEACAM1 model (heterotetramer; CbpF residues 4 - 252, CEACAM1 residues 35 - 234) against the final non-sharpened density map. **H:** Fit of atomic model in selected parts of the density map. Left: CbpF/CEACAM1 interface, right: selected glycosylation of CEACAM1. **I:** Data processing scheme with the center of reconstruction shifted towards one CEACAM1. The red arrow indicates the shifting

direction and distance. **J,K,L:** Fourier shell correlation (J), angular distribution (K), and density map colored by local resolution (L) of the cryo-EM density map of the complex with center of reconstruction shifted to one CEACAM1, originating from 330,202 particles without symmetry. **M:** Map-to-model correlation of one CEACAM1 fitted (residues 35 - 234) into the shifted density map. **N:** Fit of atomic model as of M. The black arrow indicates the position between residues 143 and 144 where the model was rejoined after building IgV and IgC2-A1 separately. The density maps in E, H, L, N, and those in F and I derived from non-uniform refinements have been sharpened with DeepEMhancer.

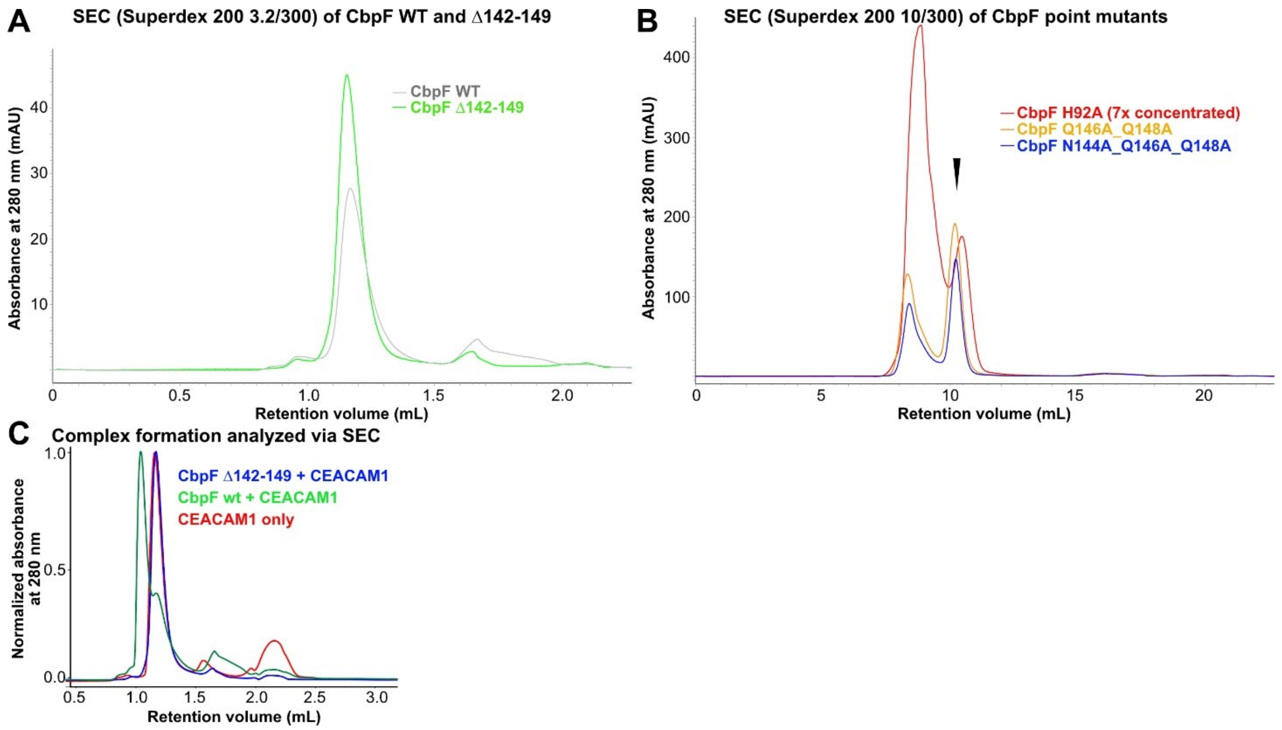

Figure S10: Purification and analysis of CbpF mutants of the CEACAM1 binding site. A: Size exclusion chromatography (Superdex 200 3.2/300) of CbpF Δ142-149 in comparison with CbpF WT. **B**: Size exclusion chromatography (Superdex 200 10/300) of CbpF mutants H92A, H92A_N99A, Q146A_Q148A, and N144A_Q146A_Q148A. The peaks at the indication (arrowhead) were used for SPR. All mutants and WT in A and B were solubilized in 25 mM MOPS-NaOH, 300 mM NaCl, 0.8% lauroylsarcosine pH 7.0. **C:** SEC profiles on a Superdex 200 3.2/300 column that show that CbpF wt (33 µg total protein amount, 1:1 ratio) forms a complex with CEACAM1, as evident by a peak shift to earlier retention volume, whereas CbpF Δ142-149 mutant (50 µg total protein amount, 1:1 ratio) does not. Note that CEACAM1 co-elutes with the CbpF mutant at the same retention volume.

Supplementary Tables

Table S1: Cryo-EM data collection, refinement and validation statistics

	**CbpF**<br>PDB 9GH4	**CbpF/CEACAM1**<br>PDB 9GH5	**1 CEACAM1 in complex**<br>PDB 9GH6
**Data collection and processing**			
Magnification	105,000		
Voltage (kV)	300		
Camera	Gatan K3 with energy filter		
Electron exposure (e–/Å^2)	79.9	52.8	
Defocus range (μm)	-1.4 – 2.6	-1.2 – 2.4	
Pixel size (Å)	0.83 (0.415 super res)	0.83	
Micrographs used	2930	6,334	
Total extracted particle images	1,054,758	1,766,853	
Refined particle images	200,809	308,537	635,781
Final particle images	46,881	211,927	330,202
Map resolution (Å)	3.76	2.67	3.04
at FSC threshold	0.143	0.143	0.143
Map resolution range (Å)	3.0 – 4.6	2.2 – 3.6	2.5 – 4.5
**Refinement**			
Refinement package	Phenix 1.21-5207		
Model resolution (Å)	3.9	2.8	3.9
at FSC threshold	0.5	0.5	0.5
Map sharpening *B* factor (Å^2)	-132.6	-115.8	-132.8
Model composition			
Non-hydrogen atoms	5454	10,383	1661
Protein residues	750	1347	200
Ligand	0	NAG: 18	NAG: 7
Water	0	3	0
B factors (min/max/mean, Å^2)			
Protein	75.16/167.36/105.25	65.23/370.39/147.09	125.60/284.48/191.15
Ligand		111.51/359.89/248.44	151.79/278.25/235.50
Water		77.35/82.83/79.88	
R.m.s. deviations			
Bond lengths (Å)	0.002	0.003	0.003
Bond angles (°)	0.599	0.715	0.607
Validation			
MolProbity score	1.53	2.08	1.77
Clashscore	7.84	9.98	8.31
Poor rotamers (%)	1.06	2.54	1.12
Ramachandran plot			
Favored (%)	97.58	96.25	95.96
Allowed (%)	2.42	3.75	4.04
Disallowed (%)	0.00	0.00	0.00

Table S2: SPR data of CbpF WT and mutants with immobilized CEACAM1.

**CbpF variant**	**Conc range (nM)**	**Rmax (RU)**	**k_a (1/Ms)**	**SE (k_a)**	**k_d (1/s)**	**SE (k_d)**	**K_D (M)**	**Chi^2 (RU^2)**
WT (Fig. 3D)	11.56 - 1479	40.54	8.13×10^{4}	1.10×10^{2}	5.01×10^{-4}	2.10×10^{-6}	6.16×10^{-9}	5.65
WT (replicate; Fig. 4I)	15.63 - 1000	20.39	1.34×10^{5}	4.20×10^{2}	8.44×10^{-4}	2.50×10^{-6}	6.29×10^{-9}	1.38
Δ142-149	15.63 - 1000	0.97	n.d.		n.d.		n.d.	
H92A	15.63 - 1000	0.39	n.d.		n.d.		n.d.	
Q146A Q148A	15.63 - 1000	0.65	n.d.		n.d.		n.d.	
N144A Q146A Q148A	15.63 - 1000	0.14	n.d.		n.d.		n.d.	

Supplementary movie legend

Supplementary Movie 1: Illustration of the structure of the CbpF/CEACAM1 complex, highlighting one binding interface. The CbpF homotrimer is shown in shades of red, and three bound CEACAM1 molecules are shown in shades of blue.
